# Humic acid improves wheat growth by modulating auxin and cytokinin biosynthesis pathways

**DOI:** 10.1093/aobpla/plae018

**Published:** 2024-03-25

**Authors:** Pramod Rathor, Punita Upadhyay, Aman Ullah, Linda Yuya Gorim, Malinda S Thilakarathna

**Affiliations:** Department of Agricultural, Food and Nutritional Science, Agriculture/Forestry Centre, University of Alberta, 9011-116St, NW, Edmonton, AB T6G 2P5, Canada; Department of Agricultural, Food and Nutritional Science, Agriculture/Forestry Centre, University of Alberta, 9011-116St, NW, Edmonton, AB T6G 2P5, Canada; Department of Agricultural, Food and Nutritional Science, Agriculture/Forestry Centre, University of Alberta, 9011-116St, NW, Edmonton, AB T6G 2P5, Canada; Department of Agricultural, Food and Nutritional Science, Agriculture/Forestry Centre, University of Alberta, 9011-116St, NW, Edmonton, AB T6G 2P5, Canada; Department of Agricultural, Food and Nutritional Science, Agriculture/Forestry Centre, University of Alberta, 9011-116St, NW, Edmonton, AB T6G 2P5, Canada

**Keywords:** *Arabidopsis*, biostimulants, GUS, humic substances, phytohormone, root growth

## Abstract

Humic acids have been widely used for centuries to enhance plant growth and productivity. The beneficial effects of humic acids have been attributed to different functional groups and phytohormone-like compounds enclosed in macrostructure. However, the mechanisms underlying the plant growth-promoting effects of humic acids are only partially understood. We hypothesize that the bio-stimulatory effect of humic acids is mainly due to the modulation of innate pathways of auxin and cytokinin biosynthesis in treated plants. A physiological investigation along with molecular characterization was carried out to understand the mechanism of bio-stimulatory effects of humic acid. A gene expression analysis was performed for the genes involved in auxin and cytokinin biosynthesis pathways in wheat seedlings. Furthermore, *Arabidopsis thaliana* transgenic lines generated by fusing the auxin-responsive *DR5* and cytokinin-responsive *ARR5* promoter to ß-glucuronidase (*GUS*) reporter were used to study the GUS expression analysis in humic acid treated seedlings. This study demonstrates that humic acid treatment improved the shoot and root growth of wheat seedlings. The expression of several genes involved in auxin (*Tryptophan Aminotransferase of Arabidopsis* and *Gretchen Hagen 3.2*) and cytokinin (*Lonely Guy3*) biosynthesis pathways were up-regulated in humic acid-treated seedlings compared to the control. Furthermore, GUS expression analysis showed that bioactive compounds of humic acid stimulate endogenous auxin and cytokinin-like activities. This study is the first report in which using ARR5:GUS lines we demonstrate the biostimulants activity of humic acid.

## Teaser

Humic acids are well known for their bio-stimulatory effects on improving plant growth and development. These beneficial effects of humic acids have been attributed to different biological functional groups and other bioactive compounds that are entrapped in their molecular structure. In this study, we demonstrate that humic acid, which is rich in hydroxyl (–OH) and carbonyl (–C=O) functional groups, improves wheat plant growth and development by eliciting the auxin and cytokinin signaling pathways.

## Introduction

Humic substances (HSs) composed of humic acid (HA), fulvic acid (FA) and ulmic acid (UA) are an important category in plant biostimulants that are known to induce plant growth and productivity. These compounds are the pool of organic carbon produced through extensive biological and chemical transformation of dead biota (Canellas *et al.*, 2015). Among different fractions of HSs, HA is the main component with a more complex structure that makes it a high molecular weight compound, which is recalcitrant to microbial degradation in soil (Ahmad *et al.*, 2018; Lumactud *et al.*, 2022). Several studies have reported the bio-stimulatory effects of humic acid in improving plant growth and development (Canellas *et al.*, 2002; Muscolo *et al.*, 2007; Schmidt *et al.*, 2007; Zandonadi *et al.*, 2007; Mora *et al.*, 2010, 2014; Trevisan *et al.*, 2010; Olaetxea *et al.*, 2019; Zanin *et al.*, 2019; Souza *et al.*, 2022). The direct benefit of humic acid application includes modifications in the roots ultrastructure (Zandonadi *et al.*, 2010; Jannin *et al.*, 2012; Nunes *et al.*, 2019), improved nutrient use efficiency (Jannin *et al.*, 2012; Zanin *et al.*, 2018), alterations in plant metabolism (Conselvan *et al.*, 2018; Roomi *et al.*, 2018; Canellas *et al.*, 2019), increase yield (Man-hong *et al.*, 2020; Shen *et al.*, 2020a) and tolerance to both biotic and abiotic stresses (Laila *et al.*, 2017; Arslan *et al.*, 2021; Faccin and Di Piero, 2022; Jiang *et al.*, 2022). The precise mechanism(s) underlying these plant growth-promoting effects of HA remains largely unknown and the limitations stem from the fact that HA is a mixture of heterogeneous compounds, and their chemical constituents (aliphatic and aromatic functional groups) vary depending on the source of origin, environmental conditions and the extraction method (Stevenson, 1994; Ritchie and Perdue, 2008). However, to a certain extent, the bio-stimulatory effects have been attributed to amino acids and a variety of functional groups such as carboxy, hydroxy, carbonyl and phenolic groups that are stabilized through hydrogen bonds and weak hydrophobic interactions within the structure of HA (Piccolo, 2001; Trevisan *et al.*, 2010; Ampong *et al.*, 2022). These bonds can be easily disrupted through the action of organic acids exudated by roots releasing several small bioactive molecules that can trigger physiological and molecular responses resulting in improved plant growth (Zandonadi *et al.*, 2007; Canellas and Olivares, 2014; Nardi *et al.*, 2017, 2018; Zanin *et al.*, 2019; Rathor *et al.*, 2023b). Humic acids are known to be active at relatively low concentrations, and it is highly unlikely that the improved plant growth in response to HA is due to the presence of macro or micro-nutrients. Instead, the positive effects on plant health appear to be due to the presence of phytohormones or their analogues within the structure or due to large-scale reprogramming of genes involved in different pathways of plant growth (Muscolo *et al.*, 1998, 1999; Mora *et al.*, 2010, 2014; Pizzeghello *et al.*, 2013; Scaglia *et al.*, 2016; Olaetxea *et al.*, 2019; Souza *et al.*, 2022).

Phytohormones are small organic molecules produced in plants. These molecules regulate many processes in plants from germination to senescence at very low concentrations (Santner *et al.*, 2009). Auxin, the first discovered phytohormone, plays a crucial role in diverse plant developmental processes throughout the lifespan of the plant (Pagnussat *et al.*, 2009; Shimizu-Sato *et al.*, 2009; Sauer *et al.*, 2013). There are several auxins and indole-acetic acid (IAA) has been studied extensively because it is the most abundant auxin in plants (Zhao, 2010). It is known to regulate many physiological and biochemical processes, including initiation and proliferation of root growth, formation of vascular tissues, apical dominance and tolerance to biotic and abiotic stresses (Muday and DeLong, 2001; Korver *et al.*, 2018). Humic acids have been shown to act as phytohormones, most commonly as auxin (Muscolo *et al.*, 1998, 1999; Russell *et al.*, 2006; Canellas *et al.*, 2011; Scaglia *et al.*, 2016; Nardi *et al.*, 2018). However, the auxin-like properties of humic acids have been debatable for a long time. The key concern has been that these compounds are applied in extremely low concentrations for bioactivity and the amount of IAA enclosed in HA at these low concentrations may be insufficient to have any physiological significance on plants (Colombo *et al.*, 2015). Existing evidence from a few studies demonstrates that HA and exogenously applied IAA induced a similar response in plants providing support to the notion that humic acid acts as auxin-like compounds. For example, Nardi *et al.* (1994) showed that when leaf explants of *Nicotiana plumbaginifolia* were treated with IAA and HA, it stimulated root growth whereas treatment using inhibitors of IAA (TIBA-2,3,5-triiodobenzoic acid and PCIB- 4-chlorophenoxy-isobutyric acid) failed to produce roots. Similarly, when leaf explants of *Nicotiana plumbaginifolia* were exposed to IAA and HA, the production of plant growth marker enzymes peroxidase and esterase was similar in both IAA and HA treatment (Muscolo *et al.*, 1993). Auxin induces stomatal opening by activation of H^+^-ATPase (reviewed by Schroeder *et al.* (2001)). It has been reported that when pea plants were treated with HA and IAA, the stomatal aperture was similar and involved the activation of phospholipase A2, which is known to be involved in auxin signalling (Russell *et al.*, 2006). However, Schmidt *et al.* (2007) demonstrated that the beneficial effect of a water-extractable fraction of humic substances obtained from sphagnum peat was independent of auxin. It failed to alter the expression of genes involved in auxin signalling in *Arabidopsis thaliana*. Moreover, it did not exhibit any differences in the GUS localization when tested using promoter–reporter transgenic lines generated with the auxin-responsive DR5 and BA3 promoters. Furthermore, Conselvan *et al.* (2018) found that exogenously applied IAA and HA exhibited different responses in metabolites of *Arabidopsis*. Therefore, the mechanism of action of humic acid seems to be highly complex and might involve different phytohormones (Zanin *et al.*, 2018; De Hita *et al.*, 2020; Souza *et al.*, 2022). Phytohormone cytokinin (Ck) plays a key role in a wide array of plant growth and developmental processes such as cell division, cell expansion, apical dominance, delay senescence and tolerance to biotic and abiotic stresses (Zwack and Rashotte, 2015; Akhtar *et al.*, 2020; Wu *et al.*, 2021). Humic acids have been shown to possess cytokinin-like activities and affect the expression of genes involved in Ck signalling (Piccolo *et al.*, 1992; Rubio *et al.*, 2009; Mora *et al.*, 2010; Pizzeghello *et al.*, 2013). However, direct evidence of HA regulating auxin and cytokinin biosynthesis pathways in wheat has not been reported to date. In this context, we hypothesized that HA might improve the shoot and root growth of wheat seedlings by eliciting endogenous auxin and cytokinin. To investigate this hypothesis, the effects of different concentrations of HA on the growth of wheat seedlings and on auxin and cytokinin responses were evaluated. This study demonstrates that HA improved the shoot and root growth by stimulating auxin and cytokinin activities in treated wheat seedlings. Gene expression analysis comparing control and HA-treated seedlings, revealed significant up-regulation of genes involved in innate pathways of auxin and cytokinin biosynthesis. Results of this study represent a major contribution to understanding the molecular mechanism through which HA in low concentration improves plant growth.

## Materials and Methods

### Fourier transform infrared (FTIR) spectroscopy

Structural analyses to investigate functional groups of HA were conducted through ATR-FTIR using the Bruker Alpha Optics system equipped with a Platinum ATR (attenuated total reflectance) accessory from Esslingen, Germany. A single-bounce ATR crystal diamond was employed during the analysis. The FTIR spectra were acquired within the wavelength range of 400–4000 cm⁻¹, and OPUS software (version 6.5) was used. Before analysing the film samples, the clean diamond ATR crystal spectrum was taken as a background spectrum. Data processing was carried out using OMNIC (Nicolet) software.

### Plant growth conditions and humic acid treatments

Wheat seeds (*Triticum aestivum* L. *var.* Parata) were obtained from the wheat breeding program at the University of Alberta. HA commercially available from Global Humic Products Ltd. Edmonton, AB was used as a biostimulant (Supplementary Table S1). Seeds were surface sterilized using 3% (*v/v*) sodium hypochlorite (NaOCl) by soaking for 2 min followed by six washing with sterile distilled water. The seeds were placed on a moistened paper towel and vertically kept in magenta (GA-7) jars (Sigma, ON, Canada) for germination in the dark at room temperature. After 5 days of growth uniform seedlings were selected and transferred in magenta jars containing 1/10 strength Hoagland basal medium with pH 5.8 (Hoagland and Arnon, 1950; Caisson labs, UT, USA) supplemented with five different concentrations of HA including 0.025% (1.2 mM carbon), 0.05% (2.4 mM carbon), 0.1% (4.8 mM carbon), 0.2% (9.6 mM carbon) and 0.4% (19.2 mM carbon) (*v/v*). The jars were placed in a plant growth room maintained at 23 °C with a 16-h light/8-h dark cycle and light intensity of 200 μmol photons m^−2^s^−1^.

### Evaluation of shoot and root growth

Seedlings grown under different concentrations of HA and control (1/10 strength Hoagland basal medium) (Hoagland and Arnon, 1950) were harvested on Days 8 and 12 after the transfer. Roots were scanned with a high-resolution scanner (Expression 12000 XL, Regent, QC, Canada). Total root length, surface area and root volume were measured with WinRhizo software (Regent, QC, Canada). The length of the longest root was recorded manually. The fresh weight of shoots and roots was recorded, then samples were oven dried at 60 °C for 5 days and dry weight was recorded. Root hairs were examined by excising 1 cm of root tissue from the middle of the primary and seminal roots (5 cm from the initiation point) at 8 days of growth. Root hair length was measured as described by Hendriks *et al.* (2022). In brief, the excised tissue previously mentioned was placed on the glass slide containing a few drops of water and gently agitated to spread the root hairs. The excised roots were examined under the Primo Star Zeiss microscope (Zeiss, ON, Canada) at 40× magnification. Root hair length was measured using the ImageJ software (Research Services Branch, NIH, Bethesda, MD, USA). The length of the longest root hairs was measured on 12 plants (two plants per biological replicate and three root samples per plant) and a total of 10 measurements were recorded for each root sample. Experiments were repeated twice with six biological replicates (*n* = 6) in each experiment at both time points and each biological replicate consisted average value of two and three plants on Days 8 and 12, respectively.

### Histochemical localization of GUS activity in transgenic plants

Seeds of *Arabidopsis thaliana* (L.) transgenic lines ARR5: GUS and DR5: GUS were obtained from ABRC (ABRC OH, USA). Seeds of wild type (Col-0; Lehle Seeds, Round Rock, TX, USA) and transgenic lines were surface sterilized using 2% (*v/v*) NaOCl by soaking for 1 min followed by four rinses with sterile distilled water and stratified at 4 °C for 2 days. Seedlings were produced as described by Rathor *et al.* (2020). In brief, sterilized seeds were placed on plates containing half-strength Murashige and Skoog (MS) medium (Fisher Scientific, ON, Canada) supplemented with 1% (*w/v*) sucrose and solidified with 0.4% (*w/v*) Gellan Gum (Fisher Scientific, ON, Canada). Plates were maintained at 22 °C with 16 h light/8 h dark cycle, with a light intensity of 200 µmol m^−2^s^−1^. Four days old seedlings were transferred into 24 well plates (Fisher Scientific, ON, Canada) containing half-strength MS medium supplemented with either/or HA (0.1%), IAA (25 μM) (Sigma, ON, Canada) and 6-benzyl aminopurine (BAP) at 10 μM (Sigma, ON, Canada). Each treatment had three biological replicates. After 24 h the seedlings were fixed in 90% cold acetone and GUS staining was performed as described by Weigel and Glazebrook (2002).

### Plant RNA isolation and quantitative real-time PCR analysis

The expression of 10 key marker genes involved in auxin and cytokinin signalling was investigated in control and HA-treated seedlings. In the auxin biosynthesis and transport, the expression pattern of *TAA1* (*Tryptophan Aminotransferase of Arabidopsis*)*, YUC1* (*YUCCA Flavin Monooxygenase*)*, TAR2.1* (*Tryptophan Aminotransferase 2.1*)*, GH3.1* (*Gretchen Hagen3.1*)*, GH3.2* (*Gretchen Hagen 3.2*)*, PIN9* (*PIN-Formed 9*) and in cytokinin biosynthesis pathway the expression of genes such as *IPT2* (*Isopentenyl Transferase 2*)*, IPT5* (*Isopentenyl Transferase 5*)*, LOG3* (*Lonely Guy3*) and *LOG5* (*Lonely Guy5*) was quantified in both shoots and roots by quantitative real–time PCR (qPCR). Wheat seeds were pregerminated, as described earlier. Five-day-old uniform seedlings were transferred in magenta jars containing 1/10 strength Hoagland medium and grown for 7 days under the previously mentioned growth conditions. After 7 days of growth, the seedlings were treated with 0.1% HA (4.8 mM carbon). This concentration was chosen as it was the intermediate concentration between lower and higher concentrations. Shoots and root tissue were collected from both control and treated plants at 24 and 120 h after HA treatment, flash frozen in liquid nitrogen and stored at −80 °C until processed. Samples were ground to a fine powder in liquid nitrogen using a pre-chilled mortar and pestle. Approximately 80–100 mg of tissue sample was used for total RNA extraction using GeneJET plant RNA purification kit (Thermo Scientific, ON, Canada). RNA quality and concentration were assessed using the Nanodrop 2000 Spectrophotometer by comparing the 260/280 and 260/230 ratios (Thermo Scientific, ON, Canada). To eliminate any possible DNA contamination, 2 µg of total RNA was treated with 2 units of RQ1 DNAse (Promega, ON, Canada). The DNAse-treated RNA was converted to cDNA, using the RevetAID cDNA Synthesis kit (Thermo Scientific, ON, Canada). The relative transcript levels were determined by qPCR, using the gene-specific primers and *β-Actin* gene as the endogenous control (Supplementary Table S2), on a Quant Studio 3 Real-Time PCR system (Applied Biosystems, ON, Canada), using iTaq SYBR Green mix (Bio-Rad, ON, Canada) (Rathor *et al.*, 2023a). The primer sequences have been previously reported (Shao *et al.*, 2017; Nguyen *et al.*, 2018). The relative expression was calculated using the delta-delta Ct method (Pfaffl, 2001), and transcript abundance was normalized to the control plants.

### Statistical analysis

The results were not significantly different between trials 1 and 2 as determined by the general linear model followed by multiple mean comparisons. Therefore, the data from the two trials (*n* = 12) were combined. Analysis of variance (ANOVA) with a confidence level of 95%, followed by the Tukey post hoc test with an error rate of 5%, was used to perform mean comparisons. Statistical analyses were performed using Minitab 19.0 (Minitab LLC, State College, PA, USA).

## Results

### Structural analysis shows two major functional groups

**Figure 1.**
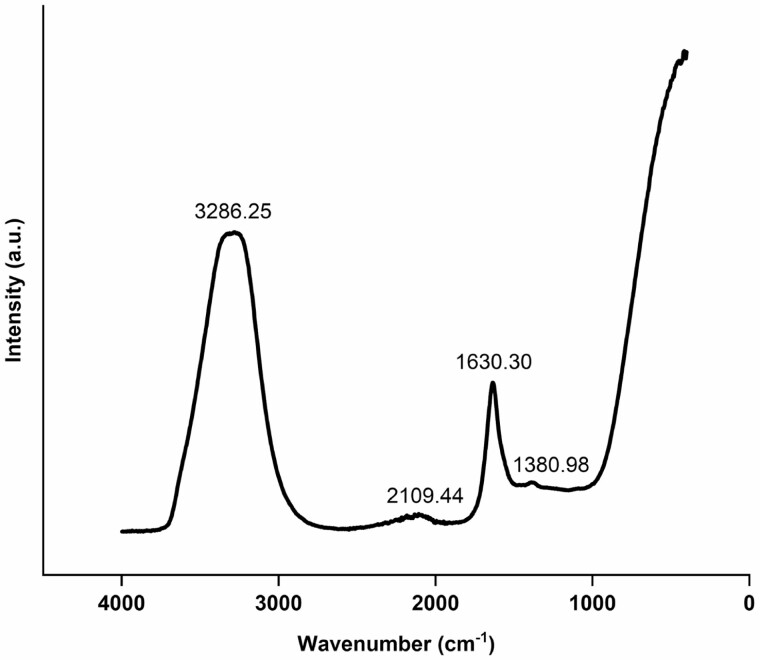
FTIR spectrum of humic acid used in this study. Au: arbitrary unit.

The FTIR spectrum of HA shown in Fig. 1, demonstrates the richness in hydroxyl (–OH) and carbonyl (–C = O) groups in HA as the predominant absorption peaks were at 3286 and 1630 cm^-1^. The absorption peaks at 3286 correspond to the –O–H stretching in alcohol and phenolic groups (Lu *et al*., 2019; Zhou *et al*., 2019). The absorption peak in the region of 1630 cm^−1^ is attributed to the –C = O of ketones, quinone and amide groups (amide I band) (Zhou *et al*., 2019; Wang *et al*., 2020). The weaker absorption peak at 1381 cm^−1^ indicates –OH deformation, –C–O stretching of phenolic –OH group, –COO asymmetric stretching and –C–H deformation of –CH_2_ and –CH_3_ groups (Zhou *et al*., 2019).

### Humic acid improved the shoot and root growth of wheat seedlings

**Figure 2.**
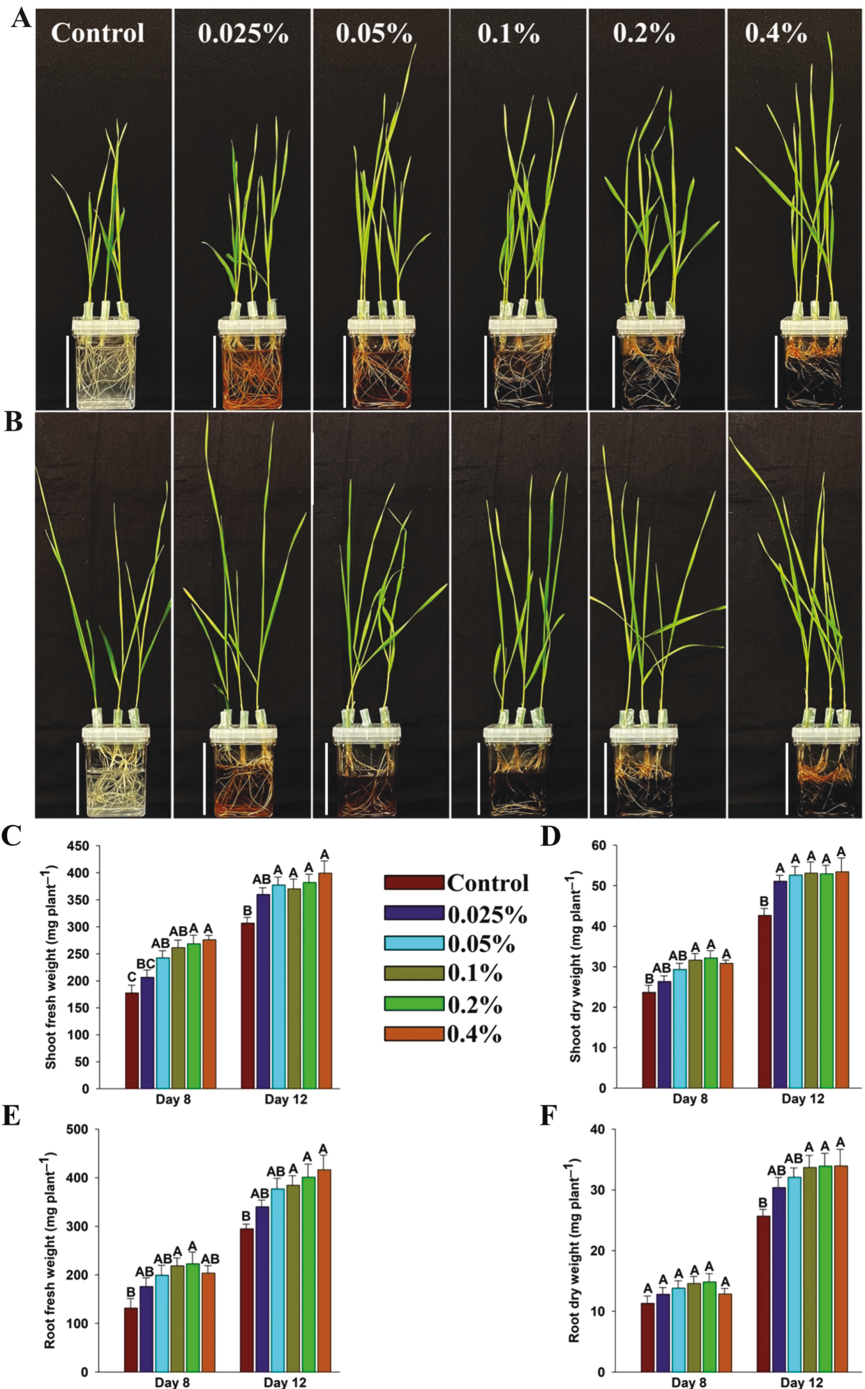
The phenotype of wheat seedlings grown under different concentrations of humic acid. Five days old seedlings were transferred in jars. (A) Seedlings were photographed 8 days after transfer into jars containing 1/10 strength Hoagland solution with or without different concentrations of humic acid. (B) Seedlings were photographed 12 days after transfer into jars containing 1/10 strength Hoagland solution with or without different concentrations of humic acid. (C) Fresh weight of shoots. (D) Dry weight of shoots. (E) Fresh weight of roots. (F) Dry weight of roots. Scale bar shows 8.5 cm. Values correspond to the means ± SE (*n* = 12). Each biological replicate consisted average value of two and three plants on days 8 and 12, respectively. Different letters above the bars represent significant differences according to Tukey’s test (*P* ≤ 0.05).

**Figure 3.**
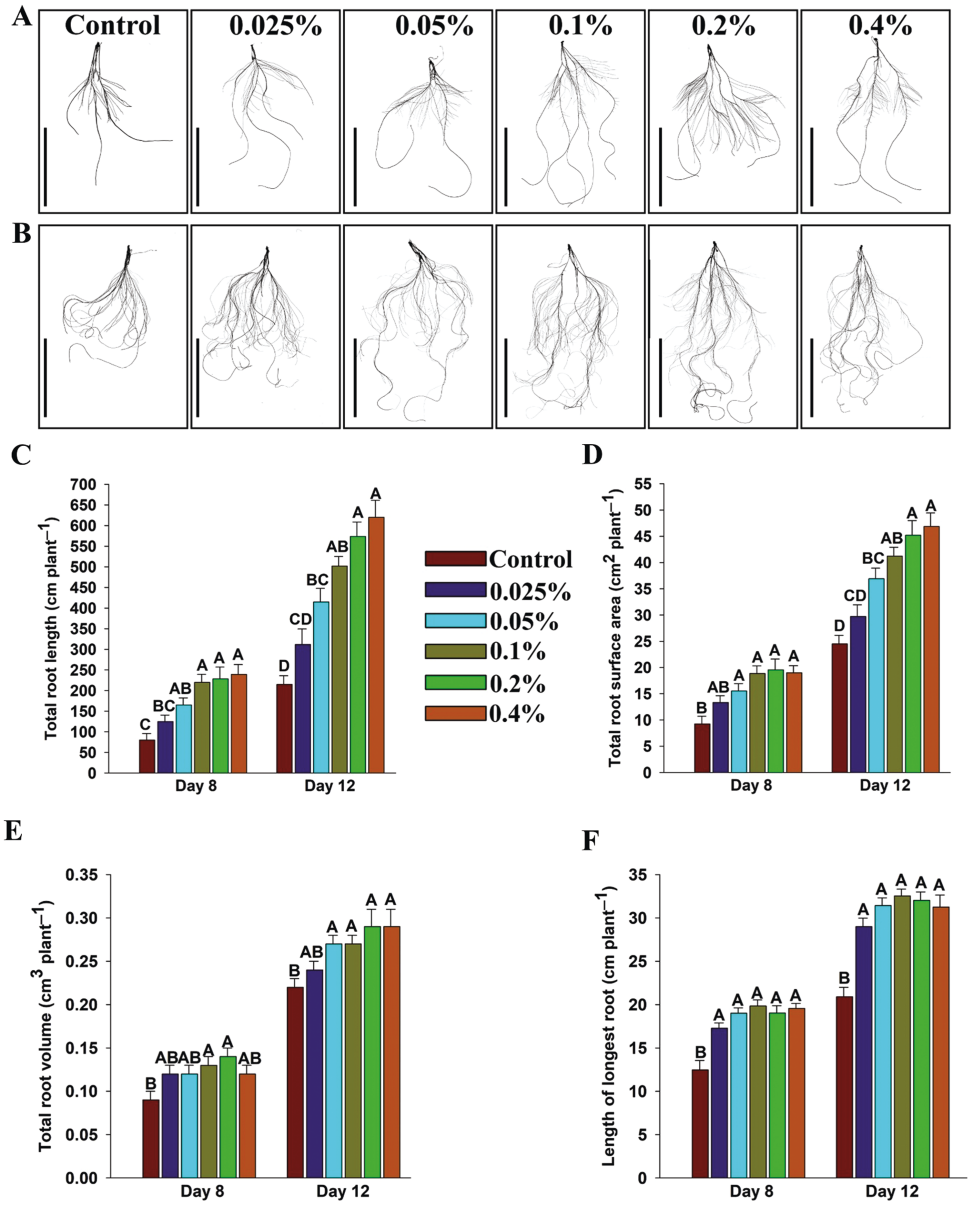
Root traits of wheat seedlings grown under different concentrations of humic acid. Five days old seedlings were transferred in jars. (A) Scanned images of roots from the seedlings 8 days after transfer into jars containing 1/10 strength Hoagland solution with or without different concentrations of humic acid. (B) Scanned images of roots from the seedlings 12 days after transfer into jars containing 1/10 strength Hoagland solution with or without different concentrations of humic acid. (C) Total root length. (D) Total root surface area. (E) Total root volume. (F) Length of the longest root. Scale bar shows 5 cm. Values correspond to the means ± SE (*n* = 12). Each biological replicate consisted average value of two and three plants on days 8 and 12, respectively. Different letters above the bars represent significant differences according to Tukey’s test (*P* ≤ 0.05).

**Figure 4.**
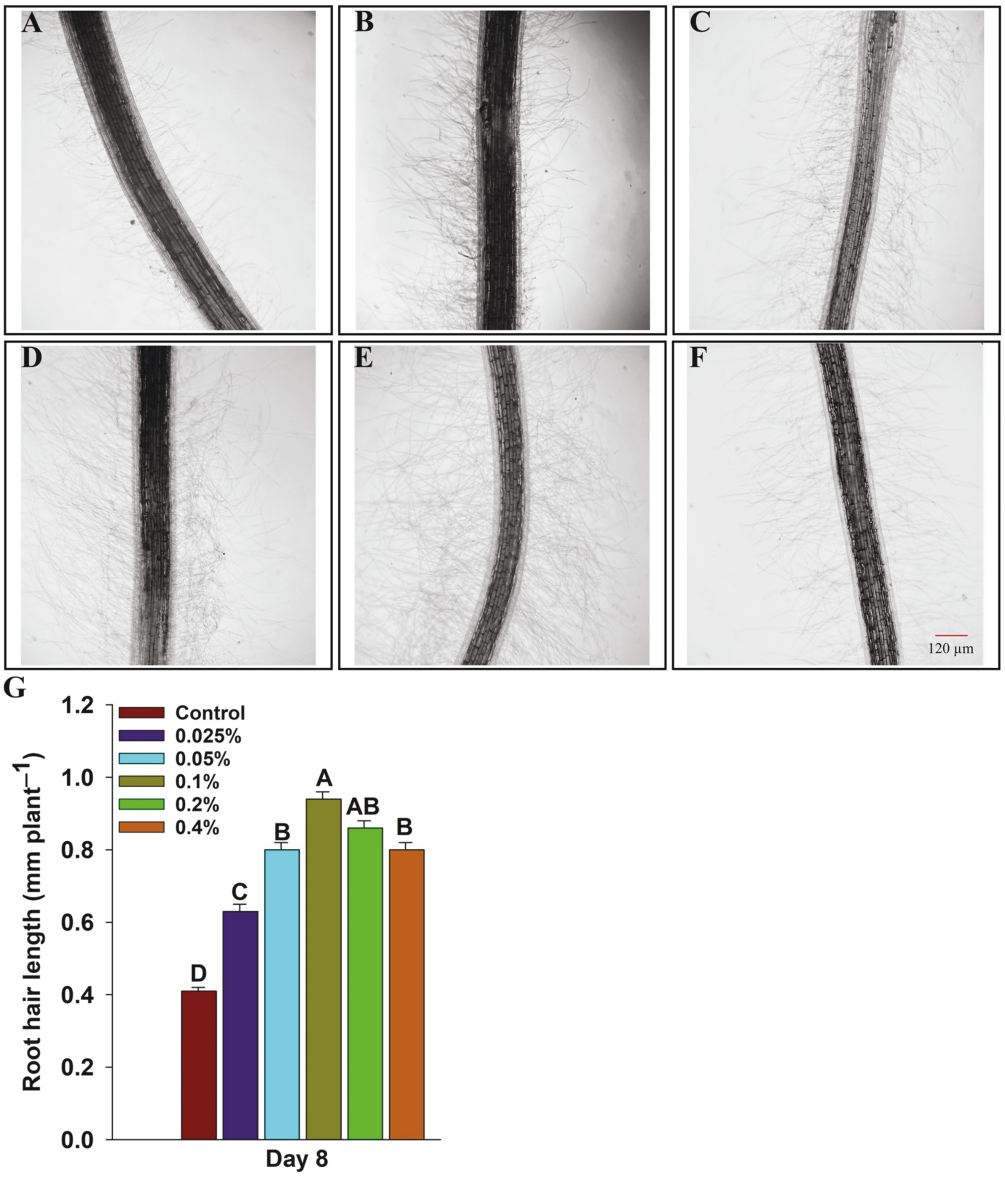
Microscopic images of root hairs and the length of longest root hairs of wheat seedlings grown under different concentrations of humic acid. Five days old seedlings were transferred in jars. Roots were excised on 8 days after transfer into jars containing 1/10 strength Hoagland solution with or without different concentrations of humic acid. In total 36 root sections in each treatment were examined and 10 measurements per root section were recorded. (A) Control. (B) 0.025% humic acid. (C) 0.05% humic acid. (D) 0.1% humic acid. (E) 0.2% humic acid. (F) 0.4% humic acid. (G) Root hair length.

Seedlings grown under different concentrations of humic acid exhibited improved shoot and root growth compared to the untreated control seedlings on both Days 8 and 12 (Figs. 2A and B and 3A and B). Wheat seedlings were grown under different HA concentrations accumulated significantly higher shoot fresh weight (16–55 and 17–30% increase on Days 8 and 12, respectively) and shoot dry weight (11-36 and 19-25% increase on Days 8 and 12, respectively) compared to the control (Fig. 2C–D). Similarly, seedlings accumulated significantly higher root fresh weight (63-106 and 15-41% increase at Days 8 and 12, respectively) and root dry weight (27-47 and 18–32% increase at Days 8 and 12, respectively) compared to the control (Fig. 2E–F). The humic acid-treated seedlings showed longer roots (38–59 and 38–55% increase at Days 8 and 12, respectively), increased total root length (55–198 and 45–188% increase at Days 8 and 12, respectively), root surface area (44–111 and 21–91% increase at Days 8 and 12, respectively) and root volume (30–54 and 4–27% increase at Days 8 and 12, respectively) compared to the control (Fig. 3C–F). Furthermore, the microscopic analysis of roots revealed that HA-treated seedlings had longer and denser root hairs compared to the untreated seedlings (Fig. 4A–F). The HA-treated seedlings showed a significant increase in root hair length (53–94% increase) compared to the control seedlings (Fig. 4G).

### Humic acid elicits the auxin and cytokinin signalling pathway

**Figure 5.**
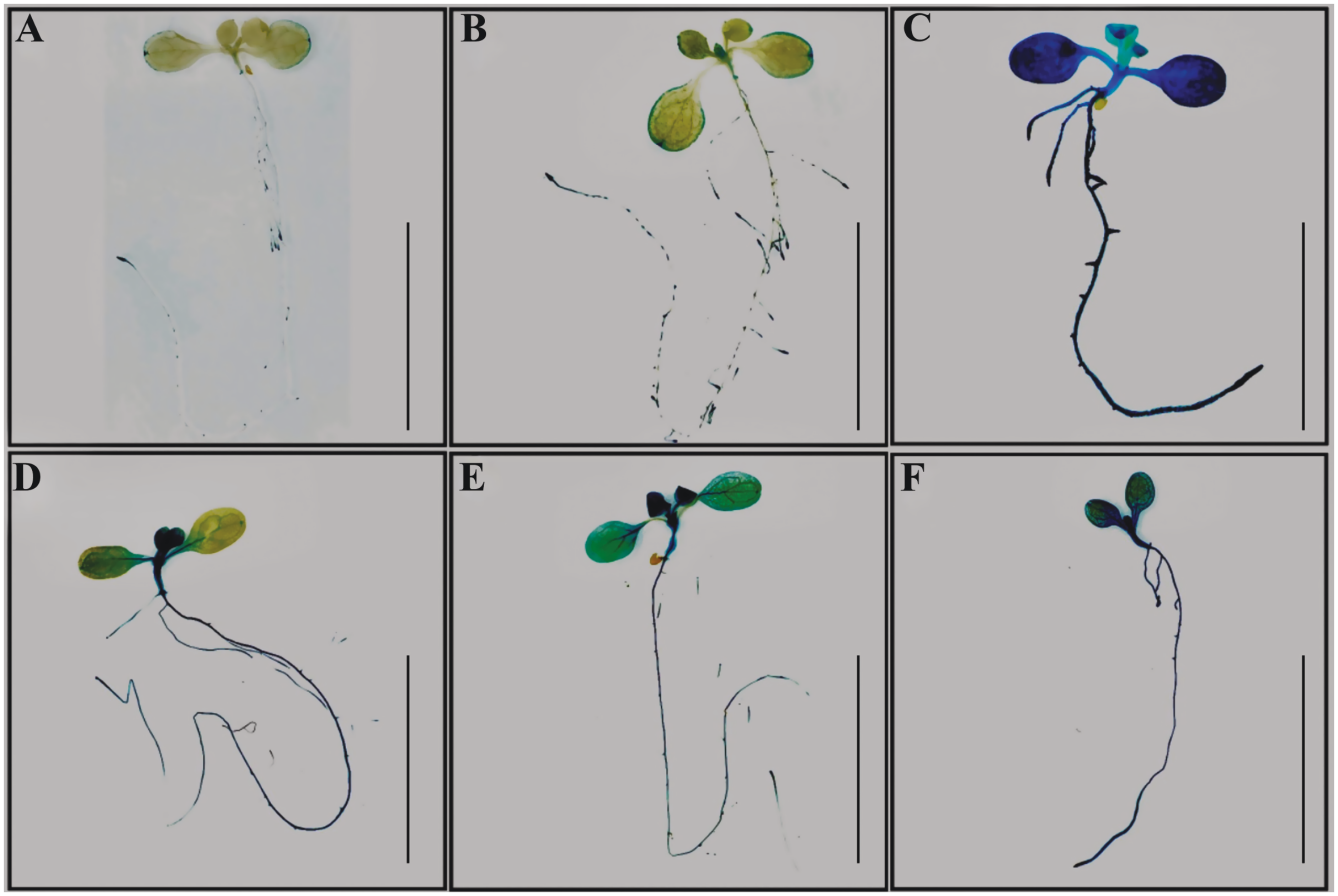
GUS staining of 6 days old transgenic *Arabidopsis* seedlings. Five days old seedlings were transferred in 24 well plate containing half-strength MS medium with or without different treatments and grown further for 24 h. (A–C) GUS staining of DR5: GUS lines at 24 h after transfer in half-strength MS media with or without treatments. (A) Half-strength MS medium. (B) Half-strength MS medium supplemented with 0.1% humic acid. (C) Half-strength MS medium supplemented with 25 μM indole acetic acid. (D–F) GUS staining of ARR5: GUS lines at 24 h after transfer in half-strength MS media with or without treatments. (D) Half-strength MS medium. (E) Half-strength MS medium supplemented with 0.1% humic acid. (F) Half-strength MS medium supplemented with 10 μM benzyl amino purine. Scale bar shows 2 cm.

The phenotypic data showed that HA improved shoot and root growth. Therefore, further investigations to assess if the cytokinin and auxin responses were altered in the presence of HA were carried out. Seedlings of *Arabidopsis* transgenic lines harbouring synthetic auxin-responsive promoter DR5 and cytokinin-responsive ARR5 promoter fused to the N-terminus of the GUS gene were used to study the GUS expression in the presence and absence of HA. The use of DR5 promoter for auxin responses has been previously reported (Dobbss *et al*., 2010; Trevison *et al*., 2010). Similarly, the *Arabidopsis* response regulator 5 (ARR5) promoter for cytokinin responses has been previously reported (D’Agostino *et al.*, 2000). It was observed that at 24 h following the exposure to HA treatment (0.1% *v/v*), the GUS activity was highly increased in DR5: GUS lines compared to the control (Fig. 5A–C). In control seedlings, the GUS activity was found at the tips of cotyledons and root tips. The GUS activity was undetectable within the newly expanding leaves and newly produced immature leaves in HA-untreated control (Fig. 5A). The GUS expression pattern was different in HA-treated seedlings compared to the control (Fig. 5B). The GUS activity was more intense in the root tips of both primary and lateral roots of HA treated seedlings compared to the untreated control. Increased levels of GUS activity were detected on the tips of cotyledons, within the newly expanding leaves, and newly produced immature leaves (Fig. 5B) compared to the HA-untreated control seedlings (Fig. 5A). In addition to auxin levels, the cytokinin levels were analysed using the cytokinin-responsive ARR5 promoter fused to the N-terminus of the GUS gene. After 24 h following the exposure to HA, the GUS activity was observed in nearly all the tissues of *Arabidopsis* seedlings under both HA-treated and untreated conditions (Fig. 5D–E). However, the GUS levels were much higher in HA-treated seedlings (Fig. 5E) compared to the HA-untreated control (Fig. 5D). The HA-treated seedlings exhibited stronger activity throughout the vasculature of the cotyledons (Fig. 5E) compared to the control seedlings (Fig. 5D). In control, seedling GUS activity was higher in the primary root and very low levels in the vasculature of the cotyledons (Fig. 5D). The expression pattern of HA-treated seedlings (Fig. 5E) was more prominent and similar to the seedlings treated with benzyl aminopurine (BAP) (Fig. 5F).

### Humic acid altered the expression of genes involved in auxin and cytokinin biosynthesis pathways

**Figure 6.**
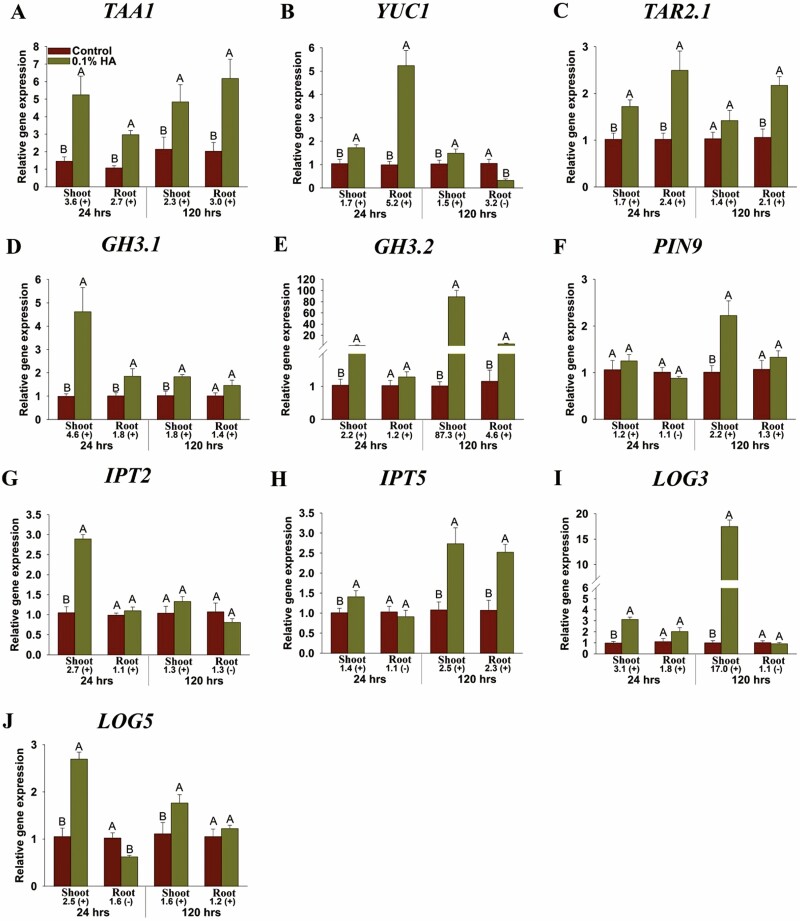
The expression level of different genes involved in auxin and cytokinin biosynthesis pathways. qPCR results demonstrate the transcript levels of several auxin and cytokinin-responsive genes in roots and shoots of control and humic acid treated seedlings at 24 h and 120 h. *β-Actin* was used as the endogenous control and transcript levels were normalized to the control. The values under the bars indicate fold differences. Values with a (+) sign represent up-regulation while values with a (−) sign represent down-regulation. Data represent mean ± SE of three independent biological replicates. (A) *Tryptophan Aminotransferase of Arabidopsis* (*TAA1*), (B) *YUCCA Flavin Monooxygenase* (*YUC1)*, (C) *Tryptophan Aminotransferase 2.1* (*TAR2.1*), (D) *Gretchen Hagen3.1* (*GH3.1*), (E) *Gretchen Hagen 3.2* (*GH3.2*), (F) *PIN-Formed 9* (*PIN9*), (G) *Isopentenyl Transferase 2* (*IPT2*), (H) *Isopentenyl Transferase 5* (*IPT5*), (I) *Lonely Guy3* (*LOG3*), and (J) *Lonely Guy5* (*LOG5*). Different letters above the bars represent significant differences according to Tukey’s test (*P* ≤ 0.05).

To elucidate the mechanism by which HA improved the shoot and root growth of wheat seedlings, the gene expression level of various standard marker genes involved in auxin and cytokinin biosynthesis pathways was studied in seedlings treated with 0.1% HA. This concentration was chosen because no significant differences were observed among 0.1 (4.8 mM carbon), 0.2 (9.6 mM carbon) and 0.4% (19.2 mM carbon) in most morphological parameters tested. In the IAA biosynthesis and transport, the expression of genes such as *TAA1, YUC1, TAR2.1, GH3.1, GH3.2* and *PIN9,* and in the cytokinin biosynthesis pathway the expression of genes such as *IPT2, IPT5, LOG3* and *LOG5* was quantified. The transcript level of these genes was significantly up-regulated (fold difference ≥1.4) in shoots and roots of HA treated seedlings at both 24 h and 120 h compared to the control seedlings (Fig. 6). In auxin biosynthetic genes, the expression level of *TAA1* (*Tryptophan Aminotransferase*) was more than 2-fold higher in HA treated seedlings compared to the control at both time points in both shoots and roots (Fig. 6A). The expression level of *YUC1* (encoding *Flavin Monooxygenase*) was significantly up-regulated at 24 h in both shoots and roots and the difference was 1.7- and 5.2-fold, respectively (Fig. 6B). The expression of this gene remained significantly up-regulated in shoots of HA treated plants at 120 h, and the difference was 1.5-fold. However, significant downregulation of *YUC1* was observed in roots at 120 h and the difference was 3.2-fold (Fig. 6B). The expression level of *TAR2.1* (*Tryptophan Aminotransferase Related*) was significantly up-regulated in both shoots and roots at 24 h, and this difference was 1.7- and 2.4-fold, respectively. The expression of this gene was significantly up-regulated in roots at 120 h, and the difference was 2.1-fold. However, no significant difference was observed in shoots at 120 h (Fig. 6C). The expression level of *GH3.1* (*Gretchen Hagen 3*) was significantly up-regulated in both shoots and roots at 24 h and this difference was 4.6- and 1.8-fold, respectively. The expression of this gene was significantly up-regulated at 120 h in shoots, and the difference was 1.8-fold. However, no significant difference was observed in roots at 120 h (Fig. 6D). The expression level of *GH3.2* (*Gretchen Hagen 3*) was strongly up-regulated in both shoots and roots at 120 h and the difference was 87.3- and 4.6-fold, respectively. A significant difference was also observed in transcript abundance of this gene in shoots at 24 h and the difference was 2.2-fold but no significant difference was recorded in roots at 24 h compared to the control seedlings (Fig. 6E). The expression level of *PIN 9* (*Pin-Formed*) was significantly up-regulated in shoots at 120 h and the difference was 2.2-fold. However, no significant difference was observed at 24 h in both shoots and roots and at 120 h in roots (Fig. 6F). In cytokinin biosynthesis, the expression level of *IPT2*, *IPT5* (*Isopentytransferase*), *LOG3* and *LOG5* (encodes *cytokinin biboside 5ʹ-monophosphate phosphoribohydrolase*) was significantly up-regulated in shoots at both 24 and 120 h and in general the expression was 2.7- and 1.3-fold for *IPT2* (Fig. 6G), 1.4- and 2.5-fold for *IPT5* (Fig. 6H), 3.1- and 17-fold for *LOG3* (Fig. 6I) and 2.5- and 1.6-fold for *LOG5* (Fig. 6J), respectively. No significant difference was observed for *IPT2* in roots at both 24 and 120 h (Fig. 6G). Significant up-regulation was observed in roots for *IPT5* at 120 h, and the difference was 2.3-fold, but no significant difference was observed at 24 h (Fig. 6H). For *LOG3* no significant difference was observed in roots at both the time points compared to the control (Fig. 6I). A significant down-regulation was found for *LOG5* in roots at 24 h, and the difference was 1.6-fold but no significant difference was found at 120 h (Fig. 6J).

## Discussion

Humic acids are known as positive regulators of plant growth and development. These compounds have been endorsed to improve the growth of several plant species, such as *Arabidopsis* (Roomi *et al.*, 2018), cucumber (Olaetxea *et al.*, 2019; De Hita *et al.*, 2020), maize (Canellas *et al.*, 2002, 2019; Zandonadi *et al.*, 2010; Ertani *et al.*, 2019; Nunes *et al.*, 2019), tomato (Jindo *et al.*, 2016), millet (Shen *et al.*, 2020b), wheat (Rathor *et al*., 2024) and canola (Jannin *et al.*, 2012). The role of humic-based compounds as phytohormone-like substances has been debated and intrigued several researchers in the past two decades. Several studies have reported that these compounds enhance root growth due to the presence of auxin-like compounds (Jannin *et al.*, 2012; Jindo *et al.*, 2016; Nardi *et al.*, 2018; Canellas *et al.*, 2019; Olaetxea *et al.*, 2019). However, positive effects on plant growth have been observed even though the levels of phytohormone were undetectable or very low enough to have any significant effect on plant growth (Jannin *et al.*, 2012; Mora *et al.*, 2012). Therefore, there is the possibility that the other bioactive compounds are entrapped in the molecular structure of HA, which could lead to the activation of endogenous phytohormone biosynthesis pathways.

Lateral roots are a key component of the root architecture as these can increase the total root length and surface area of the root system for efficient acquisition of water and nutrients (Nibau *et al.*, 2008). Activation of pericycle cells located at the xylem poles induces the production of lateral roots (Casimiro *et al.*, 2003), and auxin plays a key role in stimulating the lateral root initiation (Dubrovsky *et al.*, 2011; Lavenus *et al.*, 2013). Auxin is well known to regulate every facet of root growth and development (Roychoudhry and Kepinski, 2022). In this study, a significant increase in total root length, root surface area, root volume and length of the primary root has been observed in HA-treated seedlings compared to the control (Fig. 3). These findings are in line with previously reported studies where a significant increase in root length and number of lateral roots have been reported in HA-treated plants (Canellas *et al.*, 2002; Zandonadi *et al.*, 2007, 2010; Canellas and Olivares, 2014; Ramos *et al.*, 2015; Olaetxea *et al.*, 2019). HA has been shown to induce the formation of root hairs and the development of lateral roots and this has been attributed to phytohormone-like substances, especially auxin (Trevisan *et al.*, 2010; Zandonadi *et al.*, 2010; Mora *et al.*, 2012; Ramos *et al.*, 2015). In this study, it was found that HA-treated seedlings had longer and denser root hairs compared to the untreated control (Fig. 4). HA treatment increased the root length (Fig. 3C–F) and biomass (fresh and dry weight) of roots (Fig. 2E–F) and similar results have been reported by Nunes *et al.* (2019) where the authors showed that application of HA to maize roots increased root length and fresh weight. The increase in length can improve efficient water and nutrient absorption. Several studies have shown that HA rich in –C–O groups can enhance plant growth and development (Dobbss *et al*., 2010; Jindo *et al*., 2012; Savy *et al*., 2015). In this study, the FTIR spectrum revealed that HA was rich in –C = O groups (Fig.1). Root hair induction is shown to be negatively correlated with hydrophobic C content in HA (Canellas *et al*., 2012). In this study, FTIR analysis showed that this HA was rich in hydroxyl (–OH) functional groups, suggesting higher hydrophilic carbon content (Fig. 1). This partly explains the increased root growth observed in this study.

Auxin is a vital endogenous phytohormone that is involved in various plant growth and developmental processes, such as reshaping the root architecture, including the promotion of primary root elongation, initiation of lateral root primordia, and development of lateral roots (Aloni *et al.*, 2006; Nibau *et al.*, 2008), organogenesis (Benková *et al.*, 2003; Reinhardt *et al.*, 2003) and apical dominance (Davies, 2004; Kepinski and Leyser, 2005). In this study, it was observed that HA treatments showed increased auxin activity (Fig. 5B) and up-regulated the expression of several genes involved in auxin biosynthesis and homeostasis (Fig. 6A–F). Similar results have been reported by Souza *et al*. (2022), where the transcriptomics analysis of maize root seedlings treated using HA obtained from earthworm compost demonstrated large-scale reprogramming of genes related to auxin, cytokinin, ethylene, gibberellin, abscisic acid, jasmonic acid, salicylic acid and brassinosteroids.

**Figure 7.**
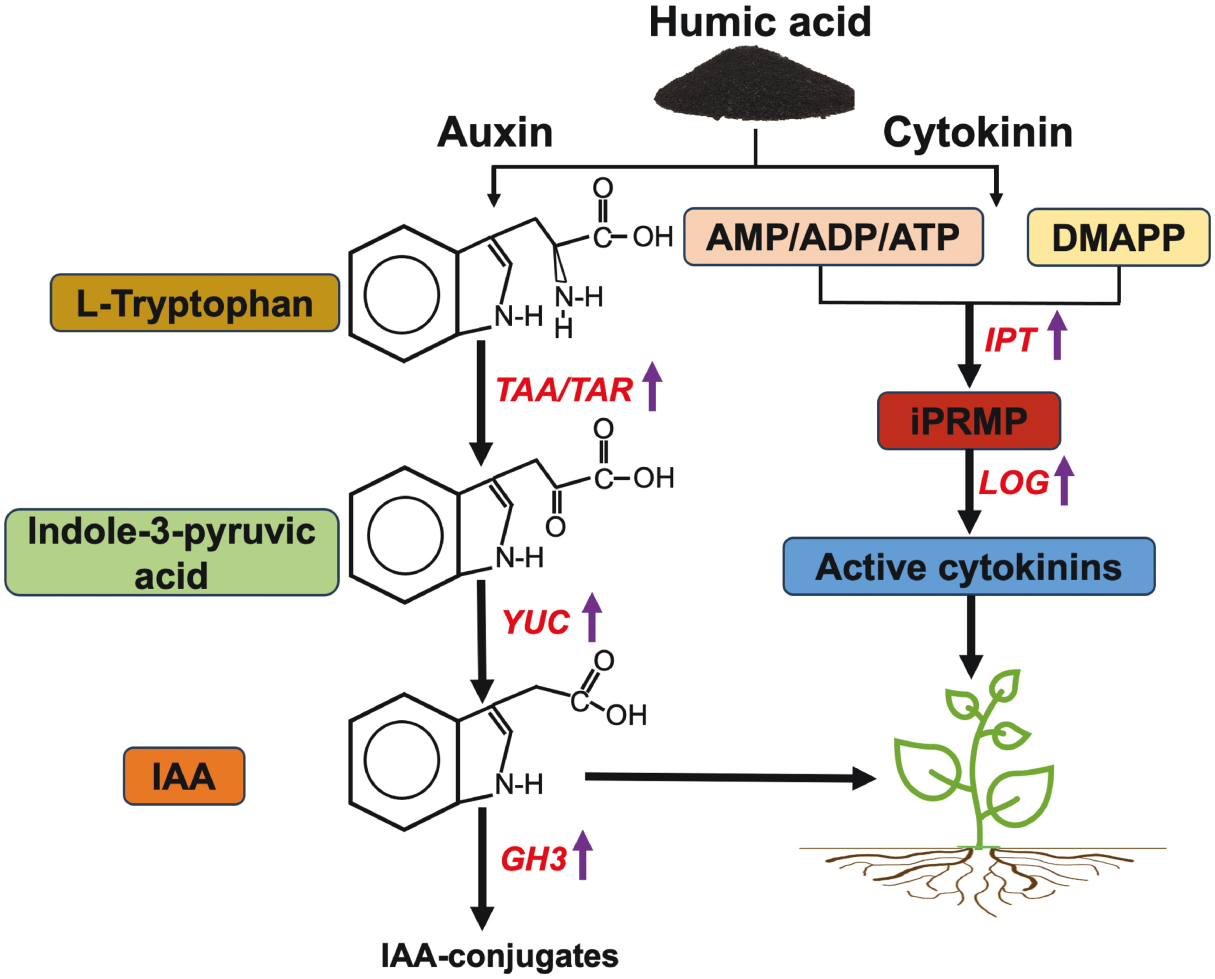
Schematic diagram of the mechanism of action of humic acid observed in this study. IAA: indole-3-acetic acid, *TAA*: *tryptophan aminotransferase of arabidopsis, TAR: tryptophan aminotransferase, YUC1: YUCCA flavin monooxygenase*, *GH3: Gretchen Hagen3,* AMP: adenosine monophosphate, ADP: adenosine diphosphate, ATP: adenosine triphosphate, DMAPP: dimethylallyl pyrophosphate, *IPT: isopentenyl transferase*, iPRMP: iP riboside monophosphate, *LOG: Lonely Guy3*.

The positive effects on root growth in response to HA treatment seem to be due to auxin-like activities of HA, as root growth is a typical auxin response (Albuzio *et al*., 1989; Casimiro *et al*., 2003; Overvoorde *et al*., 2010; Lewis *et al*., 2011; Scaglia *et al*., 2016). These findings were further corroborated by the experiments performed using the tomato and *Arabidopsis* seedlings harbouring DR5: GUS construct. The HA-treated seedlings demonstrated increased auxin activities and altered root architecture, thus providing support to the notion that HA-induced root modifications are due to enhanced auxin-like activities (Dobbss *et al*., 2010; Trevisan *et al*., 2010; Canellas *et al*., 2011). The improved root growth observed in the current study could be due to an increase in auxin-like activity, as observed by DR5: GUS and qPCR analysis of auxin-responsive genes (Figs. 5 and 6). IAA is the most extensively studied auxin in plants and it is generated by two different pathways, tryptophan (Trp) dependent and Trp independent (Woodward and Bartel, 2005). In the Trp-dependent pathway, indole-3-pyruvic acid (IPyA) pathway is the major route for auxin biosynthesis. In IPyA pathway, the first step is catalyzed by the enzyme tryptophan aminotransferase of *Arabidopsis* (TAA) or its related protein (TARs), where L-Trp is converted to IPyA and in subsequent reaction the flavin-containing monooxygenase encoded by *YUCCA* (*YUC*) gene family catalyse the conversion of the IPyA to IAA (Stepanova *et al.*, 2008, 2011; Tao *et al.*, 2008; Mashiguchi *et al.*, 2011; Won *et al.*, 2011) (Fig. 7). The loss of function mutant of *TAA* and *YUC* has demonstrated IAA deficit phenotypes (Cheng *et al.*, 2006; Stepanova *et al.*, 2008). Trp dependent pathway is the only pathway in which both catalytic steps have been biochemically characterized and is the main pathway for the biosynthesis of auxin in plants (Mashiguchi *et al.*, 2011; Won *et al.*, 2011; Ma *et al.*, 2014). In this study, it has been observed that the expression of *TAA1*, *YUC1* and *TAR 2.1* was significantly up-regulated in both shoots and roots of the HA-treated seedlings compared to the untreated control (Fig. 6A–C). *TaTAR2.1* encodes tryptophan aminotransferase and is expressed in all plant tissues. Stepanova *et al.* (2008) showed that a double mutant of *Arabidopsis* carrying a mutation in *TAA1* and *TAR2* produced abnormal phenotypes with agravitropic roots and reduced vasculature. Furthermore, the triple mutant for *TAA1*/*TAR1*/*TAR2* was seedling-lethal and failed to produce roots. A knockout mutant of wheat *TaTAR2.1* exhibited impaired lateral root growth and was defective in vegetative and reproductive development, suggesting that this is required for wheat growth and development. Furthermore, the overexpression of this gene in *Arabidopsis* increased auxin accumulation, root length, number of lateral roots, and fresh weight (Shao *et al.*, 2017). It has been shown that *TAR2*.1 is required for auxin accumulation in root primordia and lateral root initiation in *Arabidopsis* (Ma *et al.*, 2014). In the current study the expression of this gene was significantly up-regulated in HA-treated seedlings compared to the untreated control (Fig. 6C). The youngest leaves of plants show the highest capacity for *de nova* auxin biosynthesis and contain the highest levels of auxin (Ljung *et al.*, 2001). In the current study, it was found that DR5: GUS lines showed more intense GUS activity in newly emerging leaves of HA treated seedlings compared to the untreated control (Fig. 5B).

IAA levels in plants are controlled by regulating their production, transport, conjugation and degradation processes (Korasick *et al.*, 2013; Kasahara, 2016; Casanova-Sáez and Voß, 2019). In plants, IAA conjugates and catabolites strongly correlate with free IAA levels in both shoots and roots, suggesting the rapid conversion of free IAA to maintain homeostasis and prevent overaccumulation (Östin *et al.*, 1998; Kowalczyk and Sandberg, 2001). *GH3* genes are known to be induced by auxin and encode the enzyme that catalyses the conversion of free IAA to IAA-amino acid conjugates (Staswick *et al.*, 2005; Sugawara *et al.*, 2015). The expression of the *GH3* gene has been shown to be up-regulated by HA treatment in maize root seedlings (Souza *et al.*, 2022). In this study, it was found that the expression of *GH3.1* and *3.2* was significantly up-regulated in HA-treated seedlings (Fig. 6D–E). Up-regulation of IAA conjugation genes *GH3.1* and *GH3.2* suggests the negative feedback regulation due to HA-induced IAA accumulation to maintain IAA homeostasis in HA-treated seedlings (Fig. 7). In plants, auxin levels are also regulated by their transport to the different targeted tissues in the plant system. Auxin is typically synthesized in axillary buds and leaf primordia and differentially distributed throughout the plant tissues by PIN family proteins that act as efflux carriers of auxin (Sieberer and Leyser 2006; Swarup and Péret 2012). Depending on their polar localization, PINs ensure differential distribution of auxin that is vital for diverse developmental processes, including embryogenesis, tissue differentiation and organogenesis through upward, sideward, and downward transport (Sieberer and Leyser 2006; Nguyen *et al*., 2018; Manna *et al*., 2022). In this study, the expression of *PIN9* was found to be significantly up-regulated and the difference was 2.2-fold (Fig. 6F).

In this study, it was recorded that HA treatment increased the shoot biomass of wheat seedlings (Fig. 2C–D). This could be explained by the higher cytokinin activity as increased cytokinin activities are known to enhance shoot biomass (Carabelli *et al.*, 2008; Efroni *et al.*, 2013; Skalák *et al.*, 2019). Cytokinin is involved in almost all plant growth and developmental processes as it promotes cell division and cell expansion (Wu *et al.*, 2021). Previously, it has been shown that HA application on cucumber plants increased shoot fresh weight by increasing IAA levels in roots and cytokinin in shoots (Mora *et al.*, 2010, 2014). Cytokinin plays an important role in shoot development, wherein it is a prerequisite for leaf initiation. It also involves in maintaining the pluripotency of the shoot apical meristem that provides the source of stem cells for leaf initiation and aerial organ development (Wu *et al.*, 2021). The key genes involved in the cytokinin biosynthesis pathway are *IPT*s and *LOG*s that encode for isopentenyl transferase and phosphoribohydrolase, respectively (Fig. 7). In this pathway, the first-rate limiting step is catalysed by adenosine phosphate isopentenyl transferase family enzymes where cytokinin precursor isopentenyladenine (ip) nucleotides are produced from the dimethylallyl pyrophosphate (DMAPP) and adenosine phosphate (ATP/ADP or AMP) (Taya *et al.*, 1978; Hirose *et al.*, 2007). In the subsequent step, the *LOG* family genes encoding cytokinin nucleoside 5ʹ-monophosphate phosphoribohydrolase catalyse the conversion of ip Ck precursors to bioactive cytokinin (Kurakawa *et al.*, 2007). Therefore, we analysed the expression of these genes in the current study and found that the expression of both *IPT* and *LOG* family genes was significantly up-regulated in HA-treated seedlings (fold difference ≥ 1.5) compared to the untreated control (Fig. 6G–J). Exogenous application of Ck in low concentration has been shown to increase leaf size and delay senescence (Noodén *et al.*, 1979; Singh *et al.*, 1992). Overexpression of *IPT* genes has been shown to increase leaf numbers, leaf area, chlorophyll, seeds set and delay senescence (Gan and Amasino, 1995; Rivero *et al.*, 2007, 2010; Ghanem *et al.*, 2010). In this study, the expression of *IPT2* was significantly up-regulated in shoots at 24 h and *IPT5* was significantly up-regulated in both shoots and roots at 120 h (Fig. 6G–H).

Auxin and cytokinin play crucial roles in all stages of plant growth and development from seed germination to maturity. Moreover, it is well known that auxin and cytokinin have a strong antagonistic relationship (Moubayidin *et al.*, 2009; Bishopp *et al.*, 2011). Low levels of auxin restrict the cytokinin activity whereas the increase in cytokinin levels offset this inhibitory effect of auxin through inhibition of auxin activity (Kurepa *et al.*, 2019). Therefore, it is highly unlikely that treating plants with a single growth-promoting bioactive compound would lead to the accumulation of both auxin and cytokinin. As HA contains several different compounds within the macrostructure, it can induce plant growth by activating multiple pathways. However, it has been demonstrated that auxin regulates cytokinin biosynthesis in *Arabidopsis* roots as the auxin treatment induced the expression of *IPT5* and *IPT7* genes (Miyawaki *et al.*, 2004). It has been demonstrated that increased cytokinin rapidly increases auxin biosynthesis in the youngest developing leaves and roots (Jones *et al.*, 2010). Moreover, cytokinin regulates the enzymes of the Trp-dependent IAA biosynthesis pathway in the root meristem (Bielach *et al.*, 2017). Both auxin and cytokinin play an important role in the development and maintenance of shoot apical meristem, which contains the pluripotent stem cells required for plant apical growth and other plant tissues post-embryonic growth (Wu *et al.*, 2021). Auxin is required for the formation of new leaves in the apical meristem (Schaller *et al.*, 2015), whereas cytokinin is required for maintenance and initiation of leaves (Wu *et al.*, 2021). In this study, it has been found that HA alters the expression of several key marker genes involved in auxin and cytokinin biosynthesis pathways and induces the auxin and cytokinin activity, thus explaining the mechanism of the increased shoot and root growth observed in HA-treated seedlings (Fig. 7). The FTIR spectrum did not reveal the presence of auxin (Trp) and cytokinin (adenine) precursors in HA used in this study. Therefore, the possibility of other bioactive compounds present in HA that induced innate pathways of auxin and cytokinin biosynthesis pathways cannot be ruled out.

## Conclusions

The result of this study supports the hypothesis that the HA improved the shoot and root growth of wheat seedlings by activation of auxin and cytokinin biosynthesis pathways. The positive effect of HA on root architecture, such as primary root elongation, initiation and proliferation of root hairs, and root biomass can improve plant growth as alterations in root system architecture can enhance water and nutrient uptake efficiency. These findings align with previously reported studies that showed that HA altered root architecture by modulating the auxin biosynthesis pathway. This study demonstrates that results are consistent in different crops as this is the first time the molecular mechanism of HA action in improving wheat growth has been characterized. In future studies, the different bioactive components of HA should be investigated to determine the bioactive compounds responsible for modulating endogenous phytohormone biosynthesis pathways.

## Supporting Information

The following additional information is available in the online version of this article –

**Table S1.** Concentration of available elements in humic acid product.

**Table S2.** Primers used to amplify wheat genes involved in auxin and cytokinin biosynthesis pathways.

plae018_suppl_Supplementary_Tables

## Data Availability

All data supporting the findings of this study are available within the paper and within its supplementary materials. The raw data have been deposited at the Mendeley Data Repository (DOI: 10.17632/ggj4kzn3f7.1).
