# Supplementary material for: Humic acid improves wheat growth by modulating auxin and cytokinin biosynthesis pathways
Source: AoB Plants. 2024 Mar 25;16(2):plae018. doi: 10.1093/aobpla/plae018 (PMC11005776; doi:10.1093/aobpla/plae018)
Supplement: plae018_suppl_Supplementary_Tables [file plae018_suppl_supplementary_tables.pdf]

Table S1. Concentration of available elements in humic acid product.

Element	Concentration in pure (%, w:v)	Concentration in 0.1% diluted (%, w:v)
Silver (Ag)	0.00	0.0000
Aluminum (Al)	0.49	0.0005
Arsenic (As)	0.00	0.0000
Boron (B)	0.02	0.0000
Barium (Ba)	0.01	0.0000
Beryllium (Be)	0.00	0.0000
Calcium (Ca)	1.68	0.0017
Cadmium (Cd)	0.00	0.0000
Cobalt (Co)	0.00	0.0000
Chromium (Cr)	0.00	0.0000
Copper (Cu)	0.00	0.0000
Iron (Fe)	0.55	0.0005
Potassium (K)	9.08	0.0091
Lithium (Li)	0.00	0.0000
Magnesium (Mg)	0.20	0.0002
Manganese (Mn)	0.02	0.0000
Molybdenum (Mo)	0.00	0.0000
Sodium (Na)	0.33	0.0003
Nickel (Ni)	0.00	0.0000
Phosphorus (P)	0.01	0.0000
Lead (Pb)	0.00	0.0000
Sulfur (S)	0.77	0.0008
Antimony (Sb)	0.00	0.0000
Selenium (Se)	0.00	0.0000
Silicon (Si)	0.08	0.0001
Strontium (Sr)	0.02	0.0000
Titanium (Ti)	0.04	0.0000
Thallium (TI)	0.00	0.0000
Vanadium (V)	0.00	0.0000
Zinc (Zn)	0.00	0.0000
Total Nitrogen (N)	1.44	0.0014
Total Carbon (C)	48.07	0.0481

Tables S2. Primers used to amplify wheat genes involved in auxin and cytokinin biosynthesis pathway.

Gene Name	Gene/UniGene ID (Genebank)	Sequence 5' to 3'	Tm (°C)
TaTAA1-F	KM078761	GGC TGA GCA TCA GTG GTA GTA	56.3
TaTAA1-R		CCA ACG CTC ATA ACG GCC A	58.2
TaYUC1-F	CA727187	CAG TAC ACG GCG AGG TTC C	58.1
TaYUC1-R		GGC GAT CTC CAT TCC AGA GT	56.7
TaTAR2.1-F	KM078759	TGT GTC TGT CGC ATT GAA TGT C	55.7
TaTAR2.1-R		ATG TTG CTG ATC AGG AGA GAT GAC	56.6
TaGH3.1-F	AK335063	GTC CCG CTC GTG ACC TAT G	57.7
TaGH3.1-R		GAA GTG CCC GAG CTT GTG AG	58.5
TaGH3.2-F	BQ161714	ACT GGG AGC TGA TGC TCA AG	57.2
TaGH3.2-R		AGA TGG CGT AGT CCA TCA CC	56.6
TaPIN9-F	CA653575	TGT CGA TGG TGC TCA AGT TC	55.1
TaPIN9-R		TCC CAA GAA TTA CCC CTG TG	54.3
TaIPT2-2-F	GQ202267	CCG GCT CCA AAC TTT ATG TCC	56.1
TaIPT2-2-R		CGA GAC CGC GGT AGA CCT	59.1
TaIPT5-2-F	BT009376	AAG AAA CAA TCC GCA CGA AG	53.6
TaIPT5-2-R		TCG ATC GGT CAG CTT GTG TA	55.8
TaLOG3-F	BQ904276	TCG GGG TCA TTC CCA AGA CTC	58.8
TaLOG3-R		CAC CGG GCA GGG CTA TGA AG	60.6
TaLOG5-F	BM138657	GGA GGT GAA GGC CGT GTC	58.4
TaLOG5-R		TCG TAG TAC CCC TCC ACG TT	57.4
Taβ-Actin-F	LOC123120107	GGA ATC CAT GAG ACC ACC TAC	54.7
Taβ-Actin-R		GAC CCA GAC AAC TCG CAA C	56.2
